# Supplementary material for: Simultaneous Analysis of L-Carnitine and Acetyl-L-Carnitine in Food Samples by Hydrophilic Interaction Nano-Liquid Chromatography
Source: Methods Protoc. 2025 Dec 1;8(6):145. doi: 10.3390/mps8060145 (PMC12735929; doi:10.3390/mps8060145)
Supplement: Supplementary file 1 [file mps-08-00145-s001.zip › mps-3939720-supplementary.pdf]

Supplementary Information

Simultaneous Analysis of L-Carnitine and Acetyl-L-Carnitine in Food Samples by Hydrophilic Interaction Nano-Liquid Chromatography

Cemil Aydoğan [1,2,3,*], Muhammed Ercan [4] and Ziad El Rassi [5,*]

[1] Food Analysis and Research Laboratory, Bingöl University, Bingöl 12000, Türkiye; mercan@bingol.edu.tr
[2] Department of Food Engineering, Bingöl University, Bingöl 12000, Türkiye
[3] Department of Chemistry, Bingöl University, Bingöl 12000, Türkiye
[4] Graduate School of Natural and Applied Sciences, Bingöl University, Bingöl 12000, Türkiye
[5] Department of Chemistry, Oklahoma State University, Stillwater, OK 74078-3071, USA
* Correspondence: caydogan@bingol.edu.tr (C.A.); elrassi@okstate.edu (Z.E.R.)

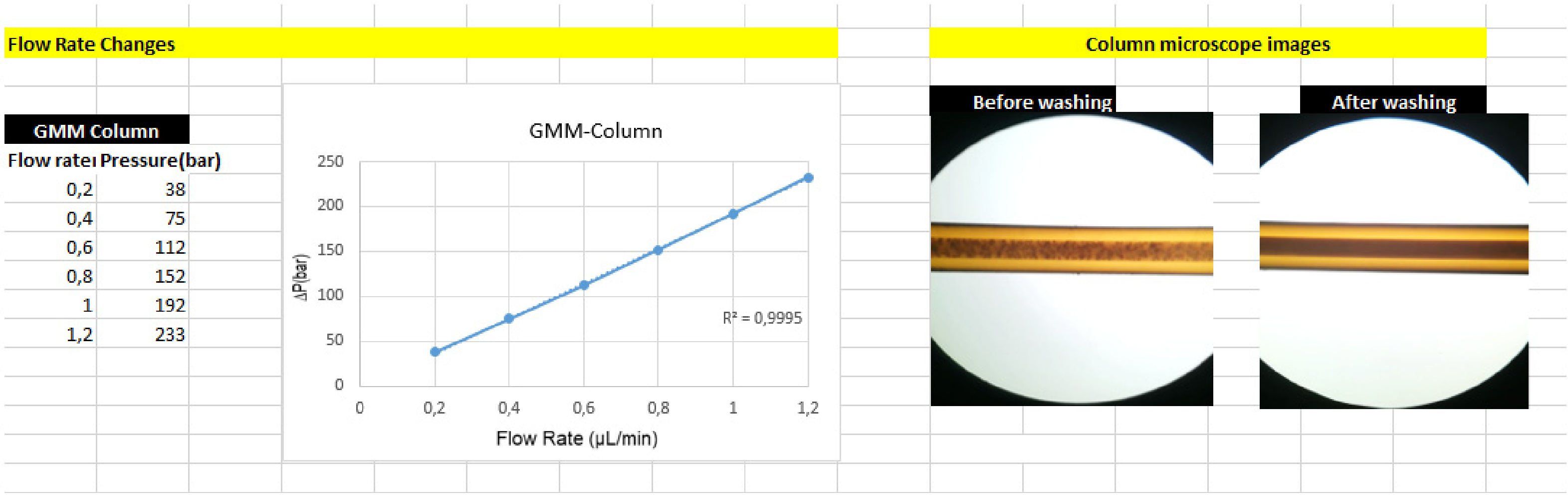

Figure S1. The prepared hydrophilic column morphology and hydrodynamic properties.

Concentration (mg/L)	Peak Area for L-CAR	Peak Area for Acetyl L-CAR
0,01	3,10	4,87
0,1	8,7	11,03
1	12,22	23,47
10	104,76	209,48
100	1004,74	2041,26
1000	11018,83	20070,19

Table S1. The calibration data for L-CAR and Acetyl-L-CAR. Chromatographic conditions: mobile phase: ACN:H_2O (92:8, v/v) with 0.1% TFA. Flow rate: 400 nL/min. Concentration range: 0.01 – 1000 mg/L (R^2): L-CAR: ~0.999 and Acetyl L-CAR: ~0.999

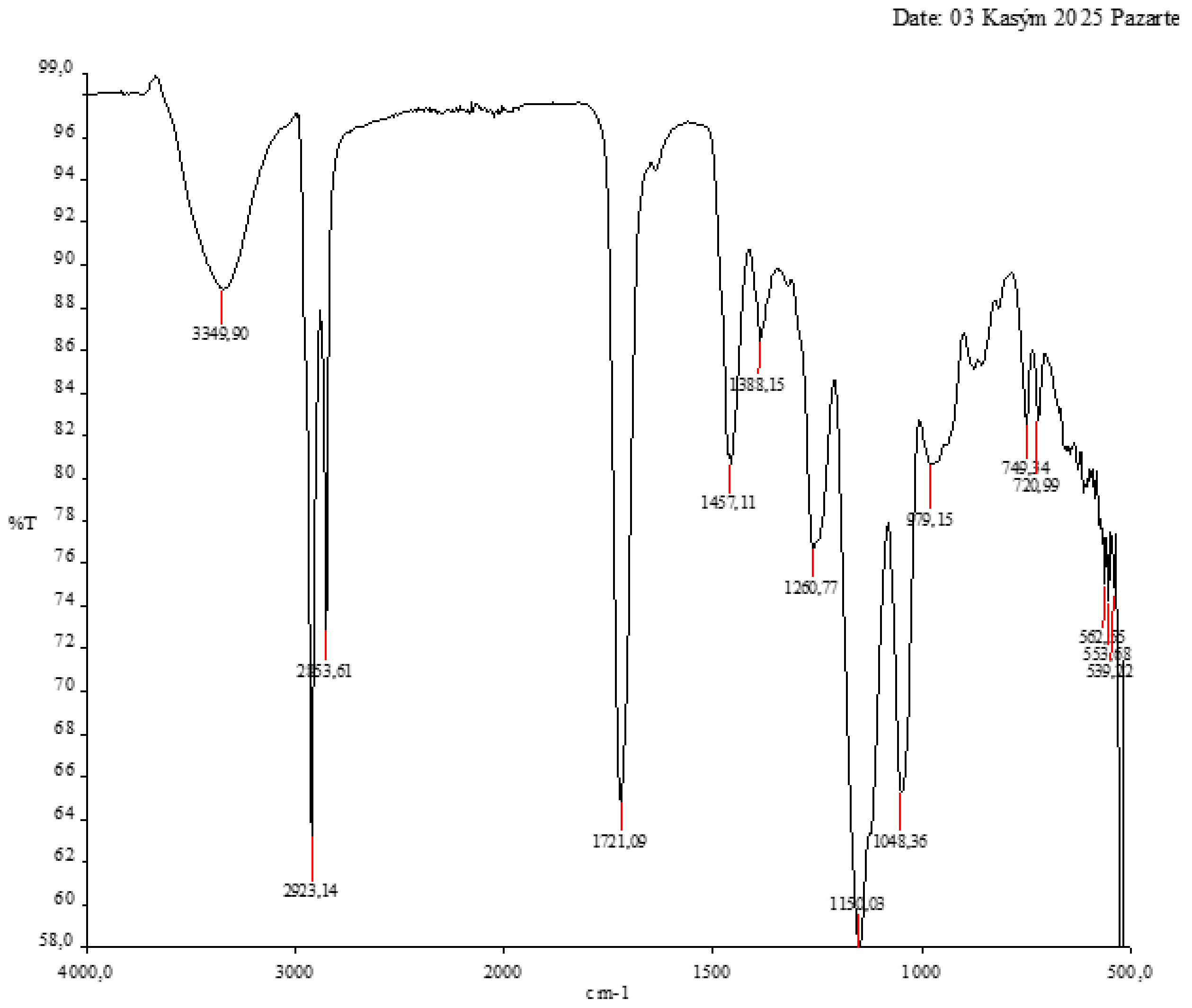

Figure S2. FT-IR spectra of the monolithic column.

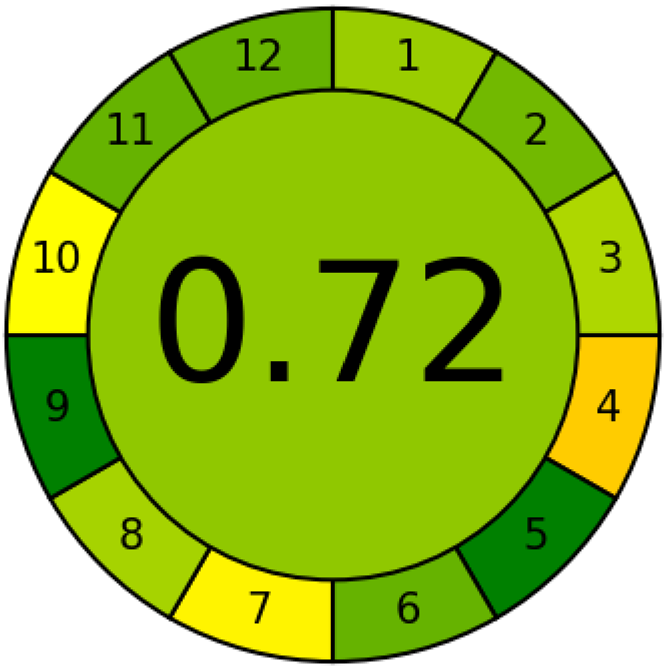

Figure S3. Analytical Greenness report (AGREE) of the developed method.
